# Regional factors as major drivers for microbial community turnover in tropical cascading reservoirs

**DOI:** 10.3389/fmicb.2022.831716

**Published:** 2022-08-18

**Authors:** Helena Henriques Vieira, Inessa Lacativa Bagatini, Guilherme Pavan de Moraes, Roberta Mafra Freitas, Hugo Sarmento, Stefan Bertilsson, Armando Augusto Henriques Vieira

**Affiliations:** ^1^Hydrobiologický ústav, Biologické cenrum AV ČR, v.v.i, .České Budějovice, Czechia; ^2^Post-graduation Program in Ecology and Natural Resources (PPGERN – CCBS), Universidade Federal de São Carlos, São Carlos, São Paulo, Brazil; ^3^Laboratory of Phycology, Department of Botany, Universidade Federal de São Carlos, São Carlos, São Paulo, Brazil; ^4^Laboratory of Microbial Processes and Biodiversity, Department of Hydrobiology, Universidade Federal de São Carlos, São Carlos, São Paulo, Brazil; ^5^Department of Aquatic Sciences and Assessment, Swedish University of Agricultural Sciences, Uppsala, Sweden

**Keywords:** ecological processes, microbial dispersion, microbial turnover, regional factors, tropical reservoirs

## Abstract

The turnover of microbial communities across space is dictated by local and regional factors. Locally, selection shapes community assembly through biological interactions between organisms and the environment, while regional factors influence microbial dispersion patterns. Methods used to disentangle the effects of local and regional factors typically do not aim to identify ecological processes underlying the turnover. In this paper, we identified and quantified these processes for three operational microbial subcommunities (cyanobacteria, particle-attached, and free-living bacteria) from a tropical cascade of freshwater reservoirs with decreasing productivity, over two markedly different dry and rainy seasons. We hypothesized that during the dry season communities would mainly be controlled by selection shaped by the higher environmental heterogeneity that results from low hydrological flow and connectivity between reservoirs. We expected highly similar communities shaped by dispersal and a more homogenized environment during the rainy season, enhanced by increased flow rates. Even if metacommunities were largely controlled by regional events in both periods, the selection had more influence on free-living communities during the dry period, possibly related to elevated dissolved organic carbon concentration, while drift as a purely stochastic factor, had more influence on cyanobacterial communities. Each subcommunity had distinct patterns of turnover along the cascade related to diversity (Cyanobacteria), lifestyle and size (Free-living), and spatial dynamics (particle-attached).

## Introduction

Spatial turnover is the variation in the species composition of communities across space and is controlled by an array of processes. In an analogy to the “big four” main processes governing population genetics, Vellend (2016) described corresponding processes that ultimately structure communities and their species composition in the metacommunity framework. These processes are selection, drift, dispersion, and speciation, the last two analogous to gene flow and mutation in population genetics, respectively.

Selection dictates the composition of communities at the local scale and comprises constraints and pressures from both environmental conditions and biotic interactions such as competition and facilitation (Ricklefs and Miller, 1990). Drift (D) on the other hand is the random fluctuations in birth and death rates in species’ populations and the loss or gain of species that occurs by chance, which may also contribute to spatial turnover in certain instances. This process is not locally or regionally determined and acts as a purely stochastic factor (Vellend, 2016). Lastly, on a regional scale, the exchange of organisms between communities (dispersion) is a component that may also influence spatial turnover. Since organisms can cross-ecosystem boundaries and move between local communities, these are not to be understood or studied as isolated entities but rather as embedded in metacommunities (Leibold et al., 2004; Logue et al., 2011). For microorganisms, such dispersion may be controlled by factors such as geographical distance or physical barriers.

Intense mobility of organisms between local communities, or homogenizing dispersion (HD), would result in communities that are more similar to each other, whereas constrained movement results in less similar communities constrained by dispersal limitation (DL; Leibold et al., 2004). However, microorganisms are small, abundant, and rapidly reproducing, thus unlikely to be under severe dispersal limitation (Bie et al., 2012; Farjalla et al., 2012). As expected, previous studies have found that enhanced dispersion may significantly influence microbial community structure (Ofiteru et al., 2010; Chase, 2010a; Langenheder and Székely, 2011; Bahram et al., 2016). Furthermore, bacteria possess several characteristics that increase their ability to cope with different environments, with the potential to override selection pressure in shaping communities. Some traits, such as flexible and modular metabolism and nutrition and widespread capacity for dormancy (Jones and Lennon, 2010), would in this way dictate their resilience to adverse conditions and disturbances.

It can be difficult to disentangle the effects of regional (dispersion) and local (selection) factors in controlling the spatial turnover of microorganisms (Logue et al., 2011). Previous studies have identified regional processes as the main drivers of microbial community variation (Finlay, 2002; Fenchel and Finlay, 2004; Nemergut et al., 2013; Zhou et al., 2013), and, in some situations, they may influence local factors. High rates of migration may, for example, insert organisms into environments that they are not well adapted to, but where they nevertheless persist over time and dominate communities due to shear mass effects (Stegen et al., 2013; Emily Graham and James Stegen, 2017).

In general, environmental variables related to selection are correlated to spatial distance, making it challenging to partition the respective role of each component. Furthermore, the most common methods used to quantify the respective roles of environmental and spatial variables shaping community turnover do not discriminate between the different ecological processes underlying it (Legendre et al., 2009; Tuomisto et al., 2012).

As a further complication, an accurate description of microbial community composition and diversity was for a long time not feasible (Rappé and Giovannoni, 2003). Recent developments and application of high-throughput sequencing of universal markers such as the 16S rRNA gene has overcome some of these bottlenecks (Stewart, 2012) but still have challenges and limitations. For example, bacterial lineages and their taxonomic marker genes may evolve at highly variable rates (Mahé et al., 2014), obscuring rational thresholds to define ecologically coherent populations or operational taxonomic units (OTUs). This may complicate the assessment of the true ability of such groups to colonize and thrive in a given habitat (Losos, 2008; Fine and Kembel, 2011).

The framework proposed by Stegen et al. (2013) can be appropriate for describing the ecological distribution patterns of organisms as abundant, widespread, and hard to culture. In brief, this method uses the *phylogenetic* turnover across habitats to define the composition and address the ecological processes underlying the observed community turnover. It assumes that lineages that are phylogenetically closely related also share traits and, consequently occupy more similar niches than distant relatives, instead of merely describing communities based on the distribution and richness of fixed cutoff OTU data (Stegen et al., 2013).

In this paper, we quantified and compared the local and regional ecological processes that drive bacterial metacommunity dynamics in a cascade of four connected freshwater reservoirs using this framework. Previous studies have been carried out on the limnology of these reservoirs (Rodgher, 2001; Sotero-Santos et al., 2006; Smith et al., 2014) but there is no comprehensive or sufficiently detailed study of microbial metacommunities aiming at understanding the biogeographical processes influencing the resident microbiota.

The cascade structure guides the dispersal of organisms from the first reservoir to the second and so on. The retention of organisms in the reservoirs is dictated by the hydraulic residence time, which varies greatly according to the rainfall regime. Given the unidirectional hydrological dispersal along these interconnected reservoirs, we can evaluate whether local factors are strong enough to influence microbial community composition along the cascade, while also quantifying the role of regional factors.

Local factors were represented by the environmental heterogeneity (trophic state gradient), while regional factors relate to the spatial distance between reservoirs and residence time. We sampled the reservoirs during both dry and rainy periods, which directly influenced the volume of water flowing through the reservoirs, the inter-system connectivity, and, hence, the environmental homogeneity/heterogeneity. We hypothesized that communities seen during the dry period would mainly be influenced by local factors, i.e., selection in response to variation in environmental factors, while communities would mostly respond to regional processes during the rainy period due to higher system connectivity and mass effects. To achieve a better resolution of the processes driving spatial turnover, we divided the bacterial community into three operational subcommunities: Cyanobacteria, Particle-attached, and Free-living bacteria expected to respond to different local environmental drivers.

## Materials and methods

### Study site

**Figure 1:**
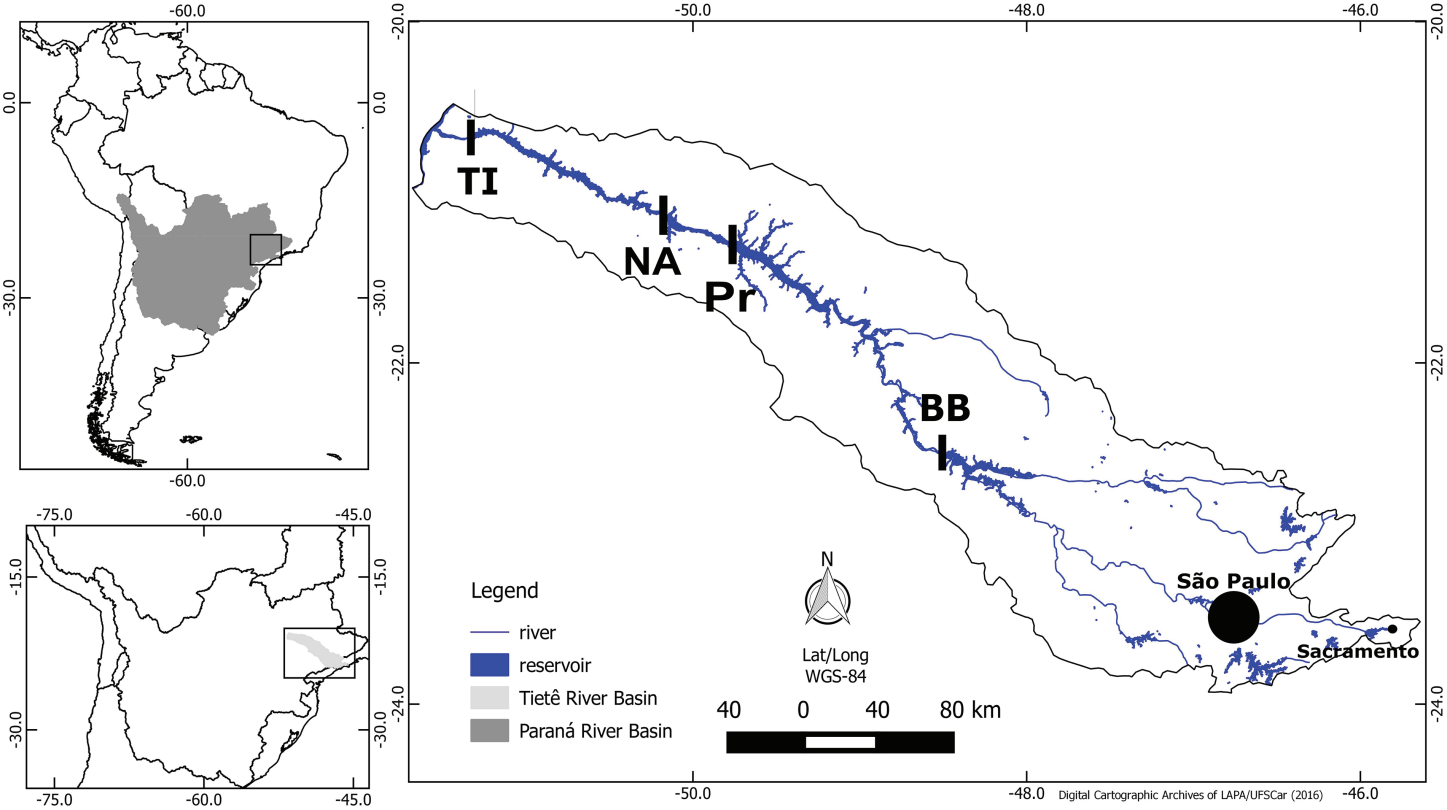
Map of Paraná Basin (topleft), Tietê River (topdown), and location of the four reservoirs sampled (right). Barra Bonita (BB), Promissão (Pr), Nova Avanhandava (NA), and Três Irmãos (TI). Modified from Freitas et al. (2018).

The four reservoirs are part of the medium-low Tietê river system, Parana River basin, São Paulo state, Brazil (Figure 1). The river cascade holds six manmade hydroelectric power reservoirs: Barra Bonita (BB), Bariri, Ibitinga, Promissão (Pr), Nova Avahandava (NA), and Três Irmãos (TI), in this order (Figure 1). Bariri and Ibitinga were not sampled in this study due to their small volume. The reservoirs are in a region surrounded by agricultural activities, resulting in inputs of large amounts of nutrients into the system, in addition to the domestic and industrial waste inputs. The water in this cascade flows toward the interior of the continent, and the anthropogenic waste load is largest in the Barra Bonita reservoir because of influences from the São Paulo metropolitan region. Thus, Barra Bonita is the most eutrophic reservoir, and the productivity decreases throughout the flow of the river because of the gradual nutrient removal/dilution effect, while the trophic state changes from hypereutrophic to eutrophic and mesotrophic (Freitas et al., 2018). Additionally, organisms are moving from Barra Bonita reservoir toward subsequent reservoirs.

### Sampling

Sampling campaigns were conducted during the dry season in May (D1), July (D2), and September (D3) of 2015, and during the rainy season in November of 2015 (R1) and January (R2) and March (R3) of 2016. Using a GPS device, we revisited specific sampling points (deepest point) of each reservoir. One liter of water from the surface of each reservoir was processed by sequential filtration, on a 3 μm polycarbonate membrane and on a 0.22 μm cellulose acetate ester membrane filter (Sterivex). Membranes were immediately frozen in liquid nitrogen after filtration and transported to the laboratory for long-term storage at-80°C. Subcommunities studied were divided into heterotrophic bacteria and Cyanobacteria. Heterotrophic bacteria were represented by Particle-attached bacteria (PA) retained on 3 μm filters and free-living bacteria (FL), retained on 0.22 μm filters. Cyanobacterial community was represented by cyanobacteria retained on 3 μm filters.

### Environmental analysis

Environmental variables such as pH, temperature, and dissolved oxygen were measured with a multiparameter sonde YSI 6600 V2 (YSI, Yellow Springs, OH, USA). Chlorophyll-a was extracted following the methods of Marker et al. (1980) and Nusch (1980), and quantified in a spectrophotometer as described by Lorenzen (1967).

Dissolved organic carbon and total nitrogen were quantified using a Shimadzu TOC-V cph analyzer, equipped with a Total Nitrogen analyzer module following the manufacturer protocol. Free phosphate and sulfate were analyzed by ion chromatography using a Dionex ICS – 1,100 system (Thermo Scientific). Water transparency was measured using a Secchi disc. All environmental variables are summarized in Supplementary information 1.

We performed a principal component analysis (PCA) with standardized environmental parameters to explore the spatial structure of environmental conditions in both rainfall periods.

### Community composition

DNA was extracted using the PowerSoil DNA Isolation kit (MoBio). Following extraction, the V3–V4 region of the 16S rRNA gene was amplified using primers 341F (5’-CCTACGGGNGGCWGCAG-3′) and 805R (5’-GACT ACHVGGGTATCTAATCC-3′) (Herlemann et al., 2011). High-throughput sequencing was carried out using the Illumina MiSeq platform. Sequences were processed using UPARSE (Edgar, 2013) for quality filtering and OTU clustering at a sequence identity ≥97%, as previously described (Logares et al., 2014; Logares, 2017). Taxonomic OTU classification was obtained with BLASTn against the SILVA 119.1 database (Zhang et al., 2000). Sequences can be accessed at NCBI: Project PRJNA411849-SRA, sequences from SRX5417418 to SRX5417491.

We assessed the phylogenetic turnover of communities (evolutionary distance between OTUs found in two communities being compared) through the quantitative framework proposed by Stegen et al. (2013). This method uses a phylogenetic tree from each community to analyze the phylogenetic turnover based on optimal OTU habitat occupation, where OTUs that are phylogenetically close are expected to share similar habitats. The method quantifies phylogenetic distance (beta-mean-nearest taxon distance, βMNTD) between each OTU in each community and between the closest relatives in the community to which it is compared (Webb et al., 2008). By subsequently shuffling species and abundances, βMNTD is compared to a randomly assembled community (null model) where selection is not influencing phylogenetic turnover.

Thus, in the first step, the method considers the deviation of the original community from the null-model (beta-nearest taxon index - βNTI), returning communities governed by selection (S), |βNTI| > 2. Communities that did not fit this first group under selection (|βNTI| < 2) went through a second randomization step using the Raup-Crick (Chase, 2010b) index modified by Stegen et al. (2013), using Bray-Curtis for each interaction (RC_bray_) to account for OTU relative abundance. At this second step, the deviation from the null model ranges from 1 to 1 and corresponds to homogenizing dispersion (RC_bray_ < −0.95), limiting dispersal (RC_bray_ > 0.95), or drift acting alone (|RC_bray_| < 0.95). For both randomization steps, the null model analysis returned a matrix of pairwise values between all four reservoirs.

We considered the ecological processes between directly connected reservoirs (Barra Bonita to Promissão, from Promissão to Nova Avanhandava, and from Nova Avanhandava to Três Irmãos, Supplementary information 2). Thus, we had three processes occurring on the cascade for each sampling time, and a total of nine processes within each rainfall period. Then, we calculated for each subcommunity the percentage of stochasticity (homogenizing and limited dispersion summed), drift, and selection, for each subcommunity.

To evaluate if community turnover was better explained by reservoir or by rainfall regime, we performed a PERMANOVA analysis based on community composition distance matrices. We used the βMNTD weighted distance, the same metric as used in null model analysis, and this index was obtained with the functions “comdistnt” and “cophenetic,” from the Picante package (Kembel et al., 2010) in R (R Core Team, 2017). We then built a non-metric multidimensional scaling (NMDS) model with communities that had significant values in PERMANOVA analysis.

All analyses were performed in computing environment *R* (R Core Team, 2017).

## Results

### Community composition

Sequencing of the 16S rRNA gene amplicons yielded 8.141.098 reads with a minimum of 9,868 reads/sample grouping into a total of 2,367 bacterial OTUs. After rarefaction, the combined dataset featured 2,112 bacterial OTUs: 39 OTUs present in Cyanobacteria; 1728 OTUs present in the particle-attached subcommunity; and 1758 OTUs present in the Free-Living subcommunity. During the dry period, the particle-attached subcommunity was represented by 1,059 OTUs and the free-living by 950 OTUs. During the rainy period, the particle-attached subcommunity was represented by 1,433 OTUs and the free-living counterpart by 1,470 OTUs.

### Environmental gradient

**Figure 2:**
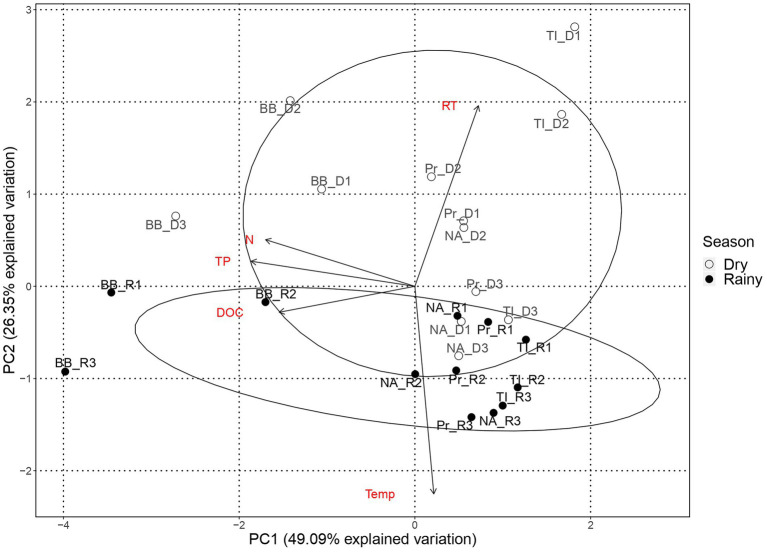
Principal component analysis of environmental variables by reservoir. Environmental variables: Residence time (RT), Temperature (Temp), Total phosphorous (TP), Dissolved organic carbon (DOC), and total nitrogen (N). Reservoirs are represented by Barra Bonita (BB), Promissão (Pr), Nova Avanhandava (NA) and Três Irmãos (TI). Black dots represent sampling points from dry period (D1, D2, and D3) and dots represent sampling points from rainy period (R1, R2, and R3).

The PC1 of the PCA analysis with environmental variables (Figure 2) reflects the reservoirs’ trophic gradient, with gradual changes in total nitrogen, total phosphorous, and DOC, the latter an indirect measure of primary production, promoting the observed spatial structure. PC2 splits the samples by season, mainly through the residence time and temperature of the water. The distribution of the samples across PC1, i.e., by trophic state, is more evident during the dry season, whereas samples are more entangled during the rainy season.

During the dry period, samples from BB and TI formed two mostly distinct groups, while samples from NA and Pr, which were the reservoirs closest to each other, overlapping in the middle of the PCA plot. Nonetheless, the PCA still reflected the order of reservoirs along the cascade during the dry season (Figure 2). During the rainy season, BB samples, from the most eutrophic reservoir, were the only samples unequivocally forming a separate and distinct group, with TI marginally separated from the more entangled group with samples from Pr and NA, once more reflecting their closer proximity. In contrast to the dry season, the order of the reservoirs in the cascade was not evident in the rainy season.

### Ecological processes

Bacterial metacommunities were analyzed separately for subcommunities of free-living, particle-attached bacteria, and cyanobacteria to investigate whether local (selection) or regional processes (homogenizing dispersion and/or dispersal limitation) or drift had the most influence on the composition of each of the three subcommunities.

PERMANOVA analyses with communities’ “βMNTD weighted distance” were performed to determine if the composition of communities was statistically dissimilar. Given the prominent separation of sampling points by season in the PCA of the environmental variables (Figure 2), we first pooled all sampling points by season for each subcommunity. We then assessed whether there was an overall dissimilarity between communities in dry and rainy periods. The PERMANOVA results showed significant (Pr(>F) < 0.05) dissimilarities between dry and rainy periods for all three subcommunities (31.98% in PA, 38.93% in FL, and 39.04% in Cyano). Subsequently, we tested if there were dissimilarities between the communities of individual reservoirs along the cascade within each season. Again, the PERMANOVA results showed significant dissimilarities (Pr(>F) < 0.05) for all three subcommunities in both seasons, except for the PA community during the rainy season.

**Figure 3:**
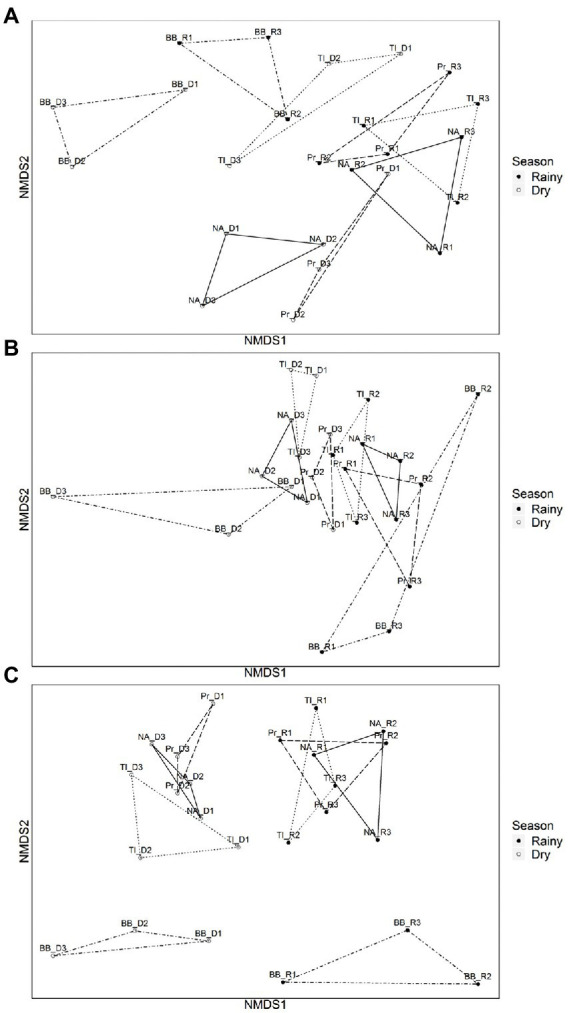
Non-metric multidimensional scaling (NMDS) ordinations with ‘βMNTD weighted’ dissimilarities of microbial communities by reservoir during dry and rainy periods. **(A)** Cyanobacterial communities (dry period: 83.83% and rainy period: 93.33%). **(B)** Particle-attached communities (dry period: 53.30% and rainy period non-significant). **(C)** Free-living communities (dry period: 71.13% and rainy period: 53.21%). PERMANOVA (Pr(>F) > 0.05). Empty dots represent sampling points from dry period (D1, D2, and D3) and filled dots represent sampling points from the rainy period (R1, R2, and R3) Reservoirs are represented by BB (Barra Bonita, connected by dot-dashed lines); Pr (Promissão connected by dashed lines); NA (Nova Avanhandava. connected by solid lines), and TI (Três Irmãos, connected by dotted lines).

The dissimilarities between seasons and between reservoirs for all three subcommunities are illustrated in the NMDS (Figures 3A–C), where samples group along NMDS1 by season, with the most evident separation for FL subcommunity, and with an overall more distinct separation of reservoirs during the dry season for the three subcommunities.

With regard to the analysis of the underlying ecological process causing this species turnover between reservoirs free-living and particle-associated bacterial communities were mostly under influence of regional processes during both rainfall regimes (Table 1). For Cyanobacteria, drift (stochastic factors) was instead the main process influencing the communities along the cascade. It was the sole identified process in drought and the main process also in the rainy period, although in the latter case, local factors (selection) and to a lesser extent also regional factors (dispersion) were significant (Table 1). The PERMANOVA analysis showed that the dissimilarities between communities were largely explained by reservoir, both in dry (83.83%) and rainy (96.33%) seasons (Figure 3A.).

**Table 1:** Ecological processes governing microbial communities between reservoirs by sampling.

		Dry			Rainy			
		D1	D2	D3	R1	R2	R3	total
Cyano	BB - Pr	D	D	D	D	D	HD	
	Pr - NA	D	D	D	D	S	D	
	NA - TI	D	D	D	D	S	D	
		Stochastic: 100%			Stochastic: 66% Regional: 11% Local: 23%			Stochastic: 83% Regional: 5% Local: 12%
PA	BB - Pr	DL	D	DL	D	DL	DL	
	Pr - NA	HD	S	D	S	HD	DL	
	NA - TI	DL	DL	D	S	D	DL	
		Stochastic: 33% Regional: 55% Local:11%			Stochastic: 22% Regional: 56% Local: 22%			Stochastic: 27% Regional: 56% Local: 17%
FL	BB - Pr	DL	S	DL	DL	DL	DL	
	Pr - NA	HD	S	HD	S	S	HD	
	NA - TI	HD	S	S	HD	S	DL	
		Regional: 56% Local: 44%			Regional: 66% Local: 34%			Regional: 61% Local: 39%

Reservoirs: Barra Bonita (BB), Promissão (Pr), Nova Avanhandava (NA), and Três Irmãos (TI). Subcommunities: Cyanobacteria (Cyano), Particle-attached (PA), and Free-living (FL). Sampling names: May-2015 (D1), July-2015 (D2), September-2015 (D3), November-2015 (R1), January-2016 (R2), and March-2016 (R3). Ecological processes driving community’s variation are presented by sampling and between representations of reservoir in the order they are on cascade. Ecological processes: Homogenizing dispersion (HD), Dispersal limitation (DL); Drift (D), and Selection (S). Percentage of ecological processes for both seasons are indicated in “total” column for each subcommunity.

The turnover of particle-attached communities was dominated by regional factors and slightly influenced by drift and local factors in both the dry and wet seasons (Table 1). PERMANOVA analysis of the dissimilarities of communities by reservoir was only significant during the dry season (59.30%, Pr(>F) > 0.05) (Figure 3B). Free-living communities were almost equally influenced by local and regional processes during the dry period, and a slightly increased influence of regional factors was seen during the rainy season, while there was no significant influence of drift in any pair of reservoirs, regardless of season (Table 1). PERMANOVA dissimilarities between FL subcommunities were explained to a greater extent by reservoir (71.13%) during the dry season than during the rainy season (56.21%) (Figure 3C).

## Discussion

The overall ambition of the study was to identify the dominant ecological processes driving the turnover of microbial communities along a cascade of freshwater reservoirs with gradually changing water quality. We expected that the lower hydrological connectivity of the reservoirs during the dry period would result in stronger gradients in water quality, which would invoke higher selection pressure on microbial communities. Conversely, during the rainy season, the elevated hydrological flow would cause environmental homogenization and increase microbial dispersion, shifting the processes controlling bacterial community composition to predominantly regional processes.

The PCA analysis of environmental variables showed that the reservoirs are more dissimilar during the dry season, confirming our first hypothesis and suggesting that the lower amount of water running through the system during the dry season led to markedly different environmental conditions along the reservoir chain (Figure 2). However, in contrast to our expectations, microorganisms seem to have been mainly under the influence of regional processes regardless of season, except for Cyanobacteria which were more strongly influenced by drift, especially during the dry season. While in contrast to our initial hypothesis, this is not altogether unexpected. All four reservoirs included in the study are eutrophic (Supplementary information 3) and feature frequent cyanobacterial blooms throughout the yearly cycle (Minillo, 2005; Rodgher et al., 2005). Blooms in these systems tend to be dominated by very few species (Minillo, 2005) and in the present study, we found a mere seven dominant genera (e.g., *Microcystis*, *Planktothrix,* and *Cylindrospermopsis*; Supplementary information 3). Our observations and results from previous studies point toward limited cyanobacterial diversity, likely caused by persistent and strong selection pressures within the system that favor these specific genera. Once such populations are established, selection pressure would weaken while drift would become increasingly important in such low-diversity communities (Chase and Myers, 2011). Likewise, if dispersion is limited, communities would be more susceptible to the effects of drift, which, in turn, would result in higher community turnover (Hubbell, 2001). We suggest that the reduced flow of water during the dry season created larger dissimilarity in environmental conditions of the reservoirs (Figure 2) along with constrained dispersion and that this led to drift being the dominant turnover process for the Cyanobacterial subcommunity.

Variation in particle-attached communities was mainly under influence of regional processes and likely depended on how easily the particles could be transported along the reservoir cascade, therefore being more strongly associated with the spatial dynamics of these particles. Particle-associated bacteria can adhere to several types of particles, such as living or dead phytoplankton cells, allochthonous detritus, polymeric organic aggregates, and clay particles, all particles but with sizes and transport characteristics that would vary both spatially (e.g., lateral transport, sinking) and temporally over seasons (Berger et al., 1996). Those differences in particle types together with the floodgate regime and distance between reservoirs could explain differences within dispersion processes (homogenized and limiting dispersion). For example, regardless of season (on 4 occasions), we observed dispersal limitations for PA subcommunities between BB and Pr. These are the reservoirs the farthest away from each other in our study and the mere distance could be the reason for the observed dispersal limitation. Furthermore, as discussed before for Cyanobacteria, a scenario of limited dispersion might favor drift, which was indeed twice identified as the dominant process for turnover of PA subcommunities between BB and Pr (Table 1).

Compared with particle-attached bacteria, the free-living community subset are usually smaller (Pedrós-Alió and Brock, 1982) and more abundant (Kirchman and Mitchell, 1982), making the free-living subcommunity more readily dispersed. Notwithstanding that particle-associated bacteria typically feature larger genome sizes than their free-living counterparts, possibly expanding their ability to adapt to variable environments and making them less sensitive to environmental change (Smith et al., 2013; Izabel-Shen et al., 2021). However, even if regional processes were overall dominating the turnover of this community subset, variation in the free-living community was also strongly influenced by selection, especially during the dry season, and this is in accordance with our hypothesis. The stronger influence of selection as compared to what was observed for cyanobacteria and particle-associated communities is an indication that free-living bacteria were more sensitive to pressures invoked by top-down or bottom-up controls or biotic interactions.

Since variables linked to the trophic state of the ecosystem had a major impact on the environmental features (Figure 2), we hypothesize that DOC from the enhanced primary production or possibly available inorganic nutrients could be major factors promoting selection. In a study conducted at the same cascade of reservoirs and during the same period that the present study was sampled, Freitas et al. (2018) found that DOC carried into the system during the rainfall period favored phytoplankton growth. As a result, the nutrient limitation due to the high uptake by phytoplankton, together with the high temperature and light intensity, led the bacterial community to allocate the carbon uptake to cell maintenance, rather than bacterial growth. Moreover, bacterial production increased under a higher concentration of labile carbon, while bacterial respiration increased under a higher concentration of recalcitrant DOC.

As these systems are highly eutrophic and display a trophic state gradient, there will be decreasing primary production along the cascade (Table 1; Figure 2). Therefore, bacterial communities profiting from and preferentially assimilating labile autochthonous DOC in upstream reservoirs would likely leave behind more recalcitrant residual carbon compounds that create a downstream gradient of increasingly recalcitrant organic carbon substrates. The bacteria that compete successfully for such labile carbon compounds would then likely be outcompeted by more locally adapted populations in downstream systems where nutrients are more scarce and where labile DOC supply from internal production is lower (Freitas et al., 2018). Such an envisioned limitation by DOC would not play the same critical role for particle-attached bacteria, because this subcommunity would mainly retrieve their substrates from the DOC released from the particles they colonize (Kent et al., 2007).

Besides the hypothesized role of DOC for subcommunities turnover, other environmental variables, such as pH and temperature, are known to influence bacterial community composition (Lindstrom et al., 2005; Lindström and Bergström, 2005; Dziallas and Grossart, 2011; Newton et al., 2011). However, in tropical environments such as the region where the studied cascade is located, the temperature is normally high throughout the year and so the amplitude is subtle when compared to temperate regions. Thus, the temperature might have an indirect effect on the bacterial community, but as an outcome of the enhanced phytoplankton activity. Nevertheless, we do not discard the possibility that other environmental variables not measured in our study, and also, trophic interactions, such as grazing and viral lysis, may have influenced bacterial composition (Verreydt et al., 2012; Declerck et al., 2013).

As a note of caution, the analytical framework proposed by Stegen et al. (2013) allows us to identify the main ecological process governing variation in microbial community composition but does not preclude that distinct processes may govern the biogeography of different groups within the same communities (Langenheder and Székely, 2011). More abundant bacteria may be under homogenizing dispersion, while less abundant ones experience constraints, such as physical barriers, grazing, and environmental pressure, causing them to be under dispersal limitation or selection. In line with this, the fractionation of the combined bacterial community into three subcommunities revealed that distinct dispersion patterns are governing the different subcommunities, even if regional factors exerted a stronger influence on the three subcommunities studied.

The predominance of different ecological processes governing each subcommunity could be linked to features such as diversity, as cyanobacteria were less rich than the other subcommunities, and diversity limitation can favor drift. Free-living communities seemed to respond more directly to environmental conditions and selection while communities attached to particles are more susceptible to differences in characteristics of the particles with biogeography shaped by spatial dynamics. Dispersion rates, generation times, and potential of colonization are also important traits determining turnover patterns and vary across bacterial taxa, which should be subject to scrutiny in future studies.

## Data availability statement

The datasets presented in this study can be found in online repositories. The names of the repository/repositories and accession number(s) can be found at: https://www.ncbi.nlm.nih.gov/, project PRJNA411849, sequences SRX5417418 to SRX5417491.

## Author contributions

HV, IB, and AV contributed to design and conception of this study. HV, IB, GM, RF, HS, and AV contributed to material preparation, samplings campaigns and data collection. HV, IB, and SB contributed to data analysis. All authors contributed to writing of the paper and approved this final version.

## Funding

Fundação de Amparo à Pesquisa (FAPESP) and Conselho Nacional de Desenvolvimento Científico e Tecnológico (CNPq) funded the project of which this study was part (processes 2011/50054–4 and 2014/14139–3). Coordenação de Aperfeiçoamento de Pessoal de Nível Superior (CAPES) and Programa de Pós-Graduação em Ecologia e Recursos Naturais (PPGERN) funded Helena Henriques Vieira, Guilherme Pavan de Moraes, and Roberta Mafra de Freitas. Grant Agency of Czech Republic (20-12496X) is funding Helena Henriques Vieira and open access fee.

## Conflict of interest

The authors declare that the research was conducted in the absence of any commercial or financial relationships that could be construed as a potential conflict of interest.

## Publisher’s note

All claims expressed in this article are solely those of the authors and do not necessarily represent those of their affiliated organizations, or those of the publisher, the editors and the reviewers. Any product that may be evaluated in this article, or claim that may be made by its manufacturer, is not guaranteed or endorsed by the publisher.
