# Supplementary material for: Regional factors as major drivers for microbial community turnover in tropical cascading reservoirs
Source: Front Microbiol. 2022 Aug 18;13:831716. doi: 10.3389/fmicb.2022.831716 (PMC9434106; doi:10.3389/fmicb.2022.831716)
Supplement: Supplementary file 1 [file Data_Sheet_1.docx]

Supplementary Inofrmation

1 - Environmental variables mean values from dry and rainy period, distance and location of reservoirs sampled from the medium-low cascade of Tietê River. A: Local factors, B: Regional factors. Environmental variables: Water transparency (Secchi), Temperature (Temp), pH, Chlorophyll-a (Chla-a), Total phosphorous (TP), phosphate (PO_4_), Dissolved organic carbon (DOC), sulphate (SO_4_), total nitrogen (TN), residence time (RT). Distance, from the first reservoir (Barra Bonita). Standard deviations are indicated after mean values.

|  |  | **Barra Bonita** | | **Promissão** | | **Nova Avanhandava** | | **Três Irmãos** | |
| --- | --- | --- | --- | --- | --- | --- | --- | --- | --- |
|  |  | **Dry** | **Rainy** | **Dry** | **Rainy** | **Dry** | **Rainy** | **Dry** | **Rainy** |
| **A** | **Secchi (m)** | 2.1 ± 1 | 0.5 ± 0.2 | 1.2 ± 0.2 | 1.3 ± 0.1 | 1.2 ± 0.2 | 1.3 ± 0.2 | 5.2 ± 2.2 | 3.2 ± 0.55 |
|  | **Temp (^o^C)** | 22.7 ± 1.6 | 27.1 ± 0.8 | 24.3 ± 1.5 | 28.0 ± 1.2 | 25.5 ± 2.0 | 28.0 ± 1.2 | 25.4 ± 1.9 | 28.6 ± 2.3 |
|  | **pH** | 7.9 ± 0.3 | 7.2 ± 0.4 | 8.7 ± 0.2 | 8.0 ± 0.6 | 9.1 ± 0.2 | 8.0 ± 0.5 | 8.3 ± 0.7 | 7.7 ± 0.4 |
|  | **Chla-a (µg.L-1)** | 22.1 ± 7.5 | 44.3 ± 28.4 | 18.9 ± 3.3 | 20.9 ± 15.1 | 16.6 ± 8 | 20.9 ± 4 | 4.7 ± 4.4 | 5.1 ± 4.3 |
|  | **TP (µM)** | 5.0 ± 1.2 | 7.1 ± 1.8 | 2. 3 ± 0.4 | 1.6 ± 0.2 | 2.0 ± 0.4 | 1.6 ± 0.7 | 1.4 ± 0.7 | 1.0 ± 0.5 |
|  | **PO_4_ (µg.L^-1^)** | 65.5 ± 29.1 | 32.8 ± 16.7 | 2.6 ± 1.8 | 7.0 ± 4.4 | 4.8 ± 2.8 | 7.0 ± 2.4 | 5.4 ± 1.8 | 12.6 ± 2.2 |
|  | **DOC (µg.C.L^-1^)** | 8.3 ± 0.4 | 13.3 ± 5.5 | 6.5 ± 1.4 | 5.9 ± 0.9 | 5.6 ± 0.2 | 5.9 ± 0.5 | 4.6 ± 0.5 | 5.0 ± 0.4 |
|  | **SO_4_ (µg.L^-1^)** | 26.9 ± 2 | 19.2 ± 4.5 | 19.1 ± 0.8 | 11.8 ± 2.6 | 16.4 ± 1.8 | 11.8 ± 1.1 | 13.4 ± 0.4 | 12.0 ± 0.6 |
|  | **TN (µM)** | 3.8 ± 1 | 4 ± 1.5 | 0.7 ± 0.1 | 0.8 ± 0.2 | 0.7 ± 0.0 | 0.8 ± 0.1 | 0.5 ± 0.1 | 0.4 ± 0.0 |
| **B** | **RT (days)** | 151 ± 30 | 47 ± 15 | 215 ± 45 | 71 ± 31 | 72 ± 22 | 71 ± 11 | 595 ± 270 | 119 ± 121 |
|  | **Distance (km)** | 0 | | 180 | | 235 | | 355 | |
|  | **Location (GPS)** | 22° 32.648’ S  048° 27.97’ W | | 21° 19.123’ S  049° 44.724’ W | | 21° 06.455’ S  050° 10.954 W | | 20° 40.110’ S  051° 16.855’ W | |

2 - Table generated with the framework used to access ecological processes driving microbial communities’ turnover along the cascade of reservoir from the medium-low Tietê River. ‘βNTI’ gives communities that were under (S) Selection (|βNTI|>2), ‘Raup-Crick’ gives the regional processes and drift governing communities variation that were not under selection at βNTI step. Ecological processes: (HD) Homogenizing dispersion (RC_bray_ < -0.95). Dispersal limitation (DL) (RC_bray_ >0.95) and (D) Drift (|RC_bray_| >0.95). Ecological processes used were those between reservoirs in the order they are on the cascade: Barra Bonita (BB), Promissão (Pr), Nova Avanhandava (NA) and Três Irmãos (TI). Subcommunities: Particle-attached (PA), Free-living (FL) and Cyanobacteria (Cyano). Sampling names: May/2015 (D1), July/2015 (D2), September/2015 (D3), November/2015 (R1), January/2016 (R2) and March/2016 (R3).

| **CYANO** | | βNTI | | |  | Raup-Crick | | |  | Ecological Process | | |
| --- | --- | --- | --- | --- | --- | --- | --- | --- | --- | --- | --- | --- |
|  |  | BB | Pr | NA |  | BB | Pr | NA |  | BB | Pr | NA |
| D1 | Pr | -1.05 |  |  | Pr | -0.84 | 0 | -0.57 | Pr | D |  |  |
|  | NA | -0.69 | -0.62 |  | NA | -0.3 | -0.57 | 0 | NA |  | D |  |
|  | TI | -0.57 | 2.7 | -0.02 | TI | 0.71 | S | 0.15 | TI |  |  | D |
|  |  | BB | Pr | NA |  | BB | Pr | NA |  | BB | Pr | NA |
| D2 | Pr | 0.8 |  |  | Pr | -0.4 | 0 | -0.34 | Pr | D |  |  |
|  | NA | -0.51 | -1.13 |  | NA | -0.2 | -0.34 | 0 | NA |  | D |  |
|  | TI | -0.31 | -0.66 | 1.46 | TI | 0.97 | 0.3 | 0.64 | TI |  |  | D |
|  |  | BB | Pr | NA |  | BB | Pr | NA |  | BB | Pr | NA |
| D3 | Pr | 0.39 |  |  | Pr | -0.73 | 0 | 0.08 | Pr | D |  |  |
|  | NA | 0.55 | 0.56 |  | NA | 0.97 | 0.08 | 0 | NA |  | D |  |
|  | TI | -0.66 | 0.19 | 0.04 | TI | 0.82 | 0.65 | -0.32 | TI |  |  | D |
|  |  | BB | Pr | NA |  | BB | Pr | NA |  | BB | Pr | NA |
| R1 | Pr | -0.39 |  |  | Pr | -0.41 | 0 | -0.5 | Pr | D |  |  |
|  | NA | -0.87 | -0.11 |  | NA | 0.24 | -0.5 | 0 | NA |  | D |  |
|  | TI | -0.31 | 1.07 | -0.99 | TI | -0.12 | -0.8 | 0.56 | TI |  |  | D |
|  |  | BB | Pr | NA |  | BB | Pr | NA |  | BB | Pr | NA |
| R2 | Pr | -1.46 |  |  | Pr | -0.26 | 0 | -1 | Pr | D |  |  |
|  | NA | 0.72 | -2.49 |  | NA | -0.08 | S | 0 | NA |  | S |  |
|  | TI | 0.36 | -1.35 | 2.72 | TI | 0.95 | -0.39 | S | TI |  |  | S |
|  |  | BB | Pr | NA |  | BB | Pr | NA |  | BB | Pr | NA |
| R3 | Pr | -1.21 |  |  | Pr | -0.95 | 0 | -0.03 | Pr | HD |  |  |
|  | NA | -1.22 | 0.37 |  | NA | 0.39 | -0.03 | 0 | NA |  | D |  |
|  | TI | -1.08 | 0.33 | -0.5 | TI | -0.93 | -1 | 0.42 | TI |  |  | D |

| **PARTICLE- ATTACHED** | | βNTI | | |  | Raup-Crick | | |  | Ecological Process | | |
| --- | --- | --- | --- | --- | --- | --- | --- | --- | --- | --- | --- | --- |
|  |  | BB | Pr | NA |  | BB | Pr | NA |  | BB | Pr | NA |
| D1 | Pr | -0.74 |  |  | Pr | 1 | 0 | -1 | Pr | DL |  |  |
|  | NA | -0.09 | 0.27 |  | NA | 0.04 | -1 | 0 | NA |  | HD |  |
|  | TI | -1.5 | 0.48 | -0.78 | TI | 1 | 1 | 1 | TI |  |  | DL |
|  |  | BB | Pr | NA |  | BB | Pr | NA |  | BB | Pr | NA |
| D2 | Pr | -1.29 |  |  | Pr | 0.2 | 0 | -0.98 | Pr | D |  |  |
|  | NA | -0.16 | 3.14 |  | NA | -0.95 | S | 0 | NA |  | S |  |
|  | TI | -0.12 | 0.14 | 0.5 | TI | 0.99 | 1 | 1 | TI |  |  | DL |
|  |  | BB | Pr | NA |  | BB | Pr | NA |  | BB | Pr | NA |
| D3 | Pr | 1.92 |  |  | Pr | 1 | 0 | -0.53 | Pr | DL |  |  |
|  | NA | -0.37 | 0.25 |  | NA | -0.17 | -0.53 | 0 | NA |  | D |  |
|  | TI | 1.35 | 1.84 | 0.55 | TI | 0.08 | 1 | 0.75 | TI |  |  | DL |
|  |  | BB | Pr | NA |  | BB | Pr | NA |  | BB | Pr | NA |
| R1 | Pr | -1.30 |  |  | Pr | 0.7 | 0 | -0.6 | Pr | D |  |  |
|  | NA | 0.42 | 2.864 |  | NA | 0.4 | -0.6 | 0 | NA |  | S |  |
|  | TI | -0.65 | 0.47 | 3.11 | TI | 1 | 1 | 1 | TI |  |  | S |
|  |  | BB | Pr | NA |  | BB | Pr | NA |  | BB | Pr | NA |
| R2 | Pr | 0.92 |  |  | Pr | 1 | 0 | -1 | Pr | DL |  |  |
|  | NA | 1.59 | 1.51 |  | NA | 1 | -1 | 0 | NA |  | HD |  |
|  | TI | 0.87 | 3.09 | 0.44 | TI | 1 | S | -0.68 | TI |  |  | D |
|  |  | BB | Pr | NA |  | BB | Pr | NA |  | BB | Pr | NA |
| R3 | Pr | 0.92 |  |  | Pr | 1 | 0 | 0.58 | Pr | DL |  |  |
|  | NA | -0.12 | -0.43 |  | NA | 1 | 0.58 | 0 | NA |  | D |  |
|  | TI | 0.29 | -0.45 | 1.974 | TI | 0.86 | 0.95 | 1 | TI |  |  | DL |

| **FREE-LIVING** | | βNTI | | |  | Raup-Crick | | |  | Ecological Process | | |
| --- | --- | --- | --- | --- | --- | --- | --- | --- | --- | --- | --- | --- |
|  |  | BB | Pr | NA |  | BB | Pr | NA |  | BB | Pr | NA |
| D1 | Pr | 1.38 |  |  | Pr | 1 | 0 | -1 | Pr | DL |  |  |
|  | NA | 0.44 | 0.48 |  | NA | -0.95 | -1 | 0 | NA |  | HD |  |
|  | TI | 0.72 | 0.35 | 0.87 | TI | 0.67 | 1 | -0.99 | TI |  |  | HD |
|  |  | BB | Pr | NA |  | BB | Pr | NA |  | BB | Pr | NA |
| D2 | Pr | 3.43 |  |  | Pr | S | 0 | -1 | Pr | S |  |  |
|  | NA | -0.35 | 2.67 |  | NA | 1 | S | 0 | N |  | S |  |
|  | TI | -2.02 | 1.96 | -5.16 | TI | -0.9 | 0 | S | TI |  |  | S |
|  |  | BB | Pr | NA |  | BB | Pr | NA |  | BB | Pr | NA |
| D3 | Pr | 1.20 |  |  | Pr | 1 | 0 | -1 | Pr | DL |  |  |
|  | NA | -0.36 | 0.56 |  | NA | 1 | -1 | 0 | NA |  | HD |  |
|  | TI | 0.39 | 4.27 | 2.71 | TI | 1 | 0.19 | -0.92 | TI |  |  | S |
|  |  | BB | Pr | NA |  | BB | Pr | NA |  | BB | Pr | NA |
| R1 | Pr | 0.12 |  |  | Pr | 1 | 0 | -1 | Pr | DL |  |  |
|  | NA | 2.01 | 2.17 |  | NA | 1 | -1 | 0 | NA |  | S |  |
|  | TI | 1.08 | 0.68 | 1.63 | TI | 1 | -0.92 | -1 | TI |  |  | HD |
|  |  | BB | Pr | NA |  | BB | Pr | NA |  | BB | Pr | NA |
| R2 | Pr | -0.17 |  |  | Pr | 1 | 0 | -1 | Pr | DL |  |  |
|  | NA | 0.82 | 2.11 |  | NA | 1 | S | 0 | NA |  | S |  |
|  | TI | -1.07 | 5.54 | 5.26 | TI | 1 | -0.9 | S | TI |  |  | S |
|  |  | BB | Pr | NA |  | BB | Pr | NA |  | BB | Pr | NA |
| R3 | Pr | -1 |  |  | Pr | 1 | 0 | -1 | Pr | DL |  |  |
|  | NA | -0.53 | -1.2 | A | NA | 0.53 | -1 | 0 | NA |  | HD |  |
|  | TI | -1 | 1.71 | -1.43 | TI | 1 | -1 | -0.46 | TI |  |  | D |

3 – Relative abundance obtained with 16S rDNA of Cyanobacteria community present on the cascade of reservoir from medium-low Tietê River. Reservoirs: BB –Barra Bonita, Pr-Promissão, NA-Nova Avanhandava and TI-Três Irmãos. Sub-communities: Cyanobacteria (Cyano), Particle-attached (PA) and Free-living (FL). Sampling names: D1-May/2015, D2-July/2015, D3-September-2015, R1-November-2015, R2-January/2016 and R3- March/2016.
